# Role and mechanism of action of leucine-rich repeat kinase 1 in bone

**DOI:** 10.1038/boneres.2017.3

**Published:** 2017-03-14

**Authors:** Weirong R Xing, Helen Goodluck, Canjun Zeng, Subburaman Mohan

**Affiliations:** 1 Musculoskeletal Disease Center, Jerry L. Pettis Memorial VA Medical Center, Loma Linda, CA, USA; 2 Department of Medicine, Loma Linda University, Loma Linda, CA, USA; 3 Department of Orthopedics, The Third Affiliated Hospital of Southern Medical University, Guangzhou, China

## Abstract

Leucine-rich repeat kinase 1 (LRRK1) plays a critical role in regulating cytoskeletal organization, osteoclast activity, and bone resorption with little effect on bone formation parameters. Deficiency of *Lrrk1* in mice causes a severe osteopetrosis in the metaphysis of the long bones and vertebrae bones, which makes LRRK1 an attractive alternative drug target for the treatment of osteoporosis and other high-turnover bone diseases. This review summarizes recent advances on the functions of the Lrrk1-related family members, *Lrrk1* deficiency-induced skeletal phenotypes, LRRK1 structure–function, potential biological substrates and interacting proteins, and the mechanisms of LRRK1 action in osteoclasts.

## Introduction

Osteoporosis, a common age-related disorder, occurs as a consequence of two major causes:^1^ low peak bone mineral density (BMD), which is typically achieved at ~age 30 years, and^2^ a high bone loss rate, which normally occurs after menopause and during the natural process of aging. Bone loss occurs with age partly when the bone resorption rate is greater than the bone formation rate. Although bone resorption is coupled with bone formation during normal physiological conditions to maintain bone homeostasis, an increased number and/or function of osteoclasts are known to contribute to excessive bone loss during disease states and aging. The processes of bone formation and bone resorption are regulated by systemic hormones, nutrition, local growth factors, and mechanical stimuli.^1,2^ A high-throughput screen aimed at the identification of the functions of over 4 500 genes led to the discovery of a leucine-rich repeat kinase 1 (LRRK1) as a critical regulator of osteoclast function and bone resorption with little effect on bone formation.^3,4^ The severe osteopetrosis phenotype in long and axial bones observed in *Lrrk1* knockout (KO) mice makes LRRK1 an ideal drug target for the prevention and treatment of osteoporotic fractures. This review summarizes recent advances on the functions of the Lrrk1-related family members, *Lrrk1* deficiency-induced skeletal phenotypes, LRRK1 structure–functional, potential biological substrates and interacting proteins, and the mechanisms of LRRK1 action in osteoclasts.

## LRRK1 family numbers

LRRK1 belongs to the ROCO family of proteins that are characterized by their unique domains including leucine-rich repeats (LRRs) and/or ankyrin repeats (ANK), a GTPase-like domain of ras of complex proteins (ROC), a C terminus of Roc domain (COR) with an unknown function, a serine/threonine kinase domain that shares sequence similarity with MAPKKK (mitogen-activated protein kinase kinase kinase), and a series of WD40 repeats in their C termini.^5–7^ In humans, there are four ROCO proteins, including MFH-amplified sequences with leucine-rich tandem repeats (MASL1), death-associated protein kinase 1 (DAPK1), LRRK1, and LRRK2. Although the ROCO family members seem to share similar structures and are ubiquitously expressed in all tissues, they do not have overlapping functions that can compensate for each other. The diverse functions of the ROCO proteins are predicted to be determined by their specific functional domains, tissue-specific expression, interacting proteins, and cross-talk with other signaling pathways in specific tissues or cell types.

### MASL1

MASL1, also known as malignant fibrous histiocytoma-amplified sequence 1, is the only ROCO protein lacking a kinase domain.^7–9^ The full-length oMASL1 protein consists of 1 053 amino acids and functions as an oncogene. The protein is overexpressed in malignant fibrous histiocytomas, gastric cancer, and hematologic malignancies.^10,11^ The chimeric MASL1 protein formed from a chromosome translocation is associated leukemic mantle cell lymphoma.^12^ *In vivo* tumorigenesis assays in nude mice have demonstrated that both MASL1 and chimeric MASL1 possess tumorigenic activity, suggesting that MASL1 is an important oncogene that regulates solid tumor and hematologic malignant cell growth.^11^ However, Kumkhaek *et al*^13^ reported that MASL1 expression was significantly increased at both messenger RNA (mRNA) and protein levels during the erythroid differentiation of CD34^+^ progenitor cells after erythropoietin stimulation, and the action was mediated via activating the Raf/MEK/ERK signaling pathway. The issue of how MASL1 regulates the proliferation of tumor cells, but modulates the differentiation of CD34^+^ progenitor cells, is unknown. Recently, a study proposed that MASL1 could first interact with heat-shock protein 60 (HSP60) to form a protein complex, then the complex could aggregate to become polymeric MASL1/HSP60 upon GDP binding to MASL1. The inactive polymeric MASL1/HSP60 complex could induce cell death. However, the polymeric MASL1/HSP60 could release active oligomeric MASL1/HSP60 upon GTP binding to MASL1. The active oligomeric MASL1/HSP60 complex stimulated cell proliferation.^11^ Thus, GTP/GDP binding to MASL1 functions as a molecular switch within the cell to regulate cell death versus proliferation.

### DAPK1

DAPK1 contains a death domain on its C terminus and a kinase domain on its N terminus, but lacks the LRR repeats.^14^ Examination of the DAPK1 kinase domain revealed that DAPK1 is a Ca2+/calmodulin-dependent kinase linked with the cytoskeleton and a mediator of apoptosis.^15^ Inhibition of DAPK1 expression suppressed apoptosis, whereas the overexpression of DAPK1 resulted in neuronal cell death.^16–18^ Mice with disruption of DAPK1 exhibited reduced renal tubule and neuron apoptosis.^19^ Recent studies found that DAPK1 could be activated by various factors, such as cellular stress and growth signaling. In normal cells, DAPK1 can activate p53, leading to p53 target gene transcription and apoptosis under death-related signaling. Simultaneously, DAPK1 could regulate cell growth by modulating TSC1/TSC2 complex formation via the mechanistic target of rapamycin (mTOR) pathway.^20,21^ Phosphorylation of TSC2 at serine 939 by DAPK1 disrupts the dimerization of TSC1 with TSC2, leading to increased cell growth and protein synthesis, and thereby maintaining a homeostatic balance between survival and death signaling. In p53 mutant cells, however, DAPK1 could not activate the p53-mediated death signaling pathways, resulting in a shift of function from apoptosis toward activation of the growth pathways.^21,22^ Unlike other ROCO family members, GTP binding to the ROC domain of DAPK1 negatively regulates the kinase activity via inducing its inhibitory auto-phosphorylation.^23,24^

### LRRK2

LRRK2 is one of the most studied proteins among the ROCO family proteins. Mutations in *Lrrk2* have been associated with autosomal-dominant Parkinson’s disease (PD), a neurodegenerative disorder with symptoms of resting tremor, postural instability, muscle rigidity, and bradykinesia.^25,26^ The *Lrrk2* gene encodes a large multi-domain protein of 2 527 amino acids. A mutation of G2019S in the kinase domain of LRRK2 has been shown to elevate its kinase activity, GTP binding, and contributed to PD, whereas other mutations identified in patients with PD had no effect on the kinase activity.^27–31^ Patients carrying the G2019S mutation showed neurodegeneration, including loss of dopaminergic neurons and accumulation of Lewy bodies in the cytoplasm.^32^ Transgenic mice that overexpress the G2019S mutant LRRK2 also exhibited neuronal degeneration.^33^ Greggio *et al*^34,35^ found that a decrease in the kinase activity of LRRK2, either by synthetic mutations in the kinase domain or by abolishing GTP binding to LRRK2, relieved the toxicity resulting from pathogenic mutations. Mice lacking the LRRK2 protein showed an early-onset increase in number and size of secondary lysosomes in kidney cells and lamellar bodies in lung cells, whereas mice expressing the LRRK2 kinase-dead mutant from an endogenous locus displayed similar early-onset pathophysiological changes in the kidneys but not in the lungs. Tong *et al* reported that the loss of *Lrrk2* impaired protein degradation pathways, resulting in an accumulation of α-synuclein, and led to marked increases in apoptotic cell death, inflammatory responses, and oxidative damage in the kidneys, but not to neurodegeneration or neuropathological changes in the brain of aged mice.^36^ Mutations in the ROC domain (K1347A and T1348N) of LRRK2 prevented GTP binding and reduced kinase activity as well.^37^ Interestingly, mice with the disruption of *Lrrk2* exhibited no obvious skeletal phenotypes.^3^ The discrepant phenotypes among different LRRK2 mutant mice strongly suggested that other structural domains besides the kinase domain also function via forming scaffold complexes, GTP/GDP switches, or protein/protein interactions.

### LRRK1

**Figure 1:**
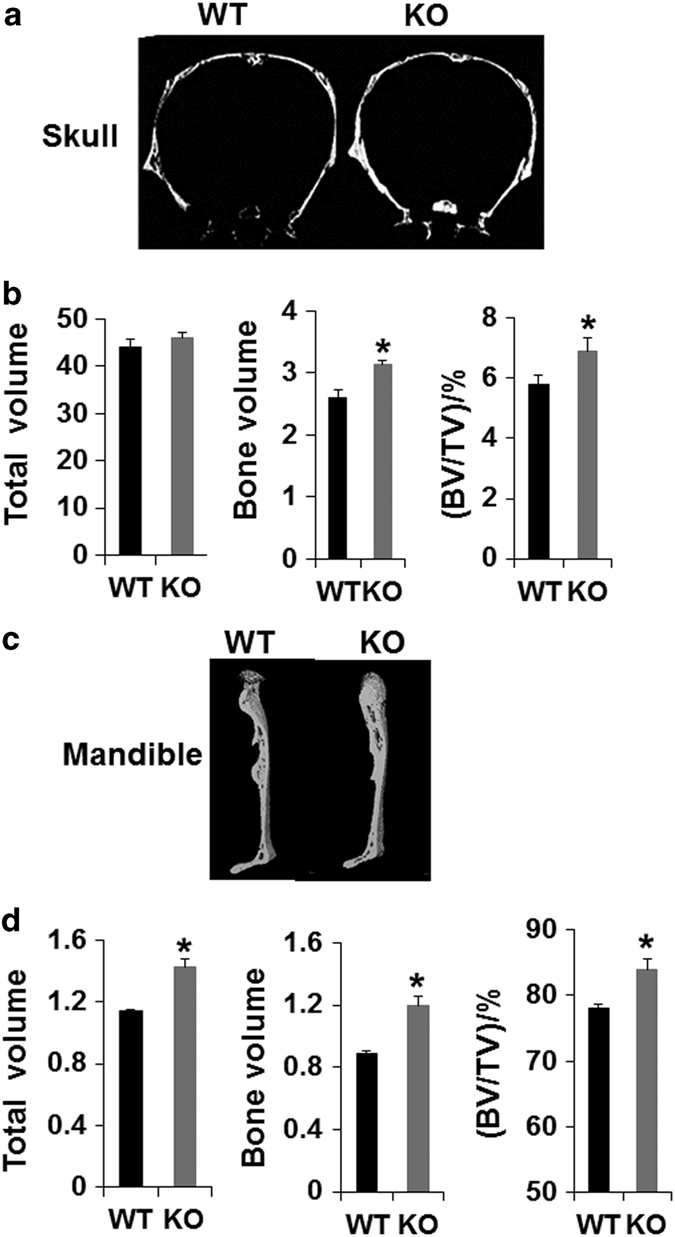
LRRK1 regulation of osteoclast function is different in membranous versus endochondral bone. Our previous studies showed that mice with disruption of Lrrk1 exhibited a severe osteopetrosis phenotype in the trabecular fraction of the metaphases of long bones and vertebrae, which form by endochondral bone formation. To address the question of whether a lack of LRRK1 also influences skeletal sites that mainly form through the intramembranous route, we analyzed calvarial and mandibular bones of 6-week-old male *Lrrk1* KO mice and control WT littermates (*N*=4 pairs) by micro-CT. In contrast to markedly elevated trabecular BV/TV in long bones and vertebrae that had two- to fivefold increases in bone volume and bone mineral density,^3^ deficiency of *Lrrk1* had only a mild effect on the skull. The calvarias from *Lrrk1* KO mice had normal TV but 20% higher BV and 17% higher BV/TV than control mice (**a, b**). For the mandible, TV, BV, and BV/TV in *Lrrk1* KO mice were increased 25%, 34%, and 7%, respectively (**c**, **d**). *P<0.05.

The human *Lrrk1* gene is located on chromosome 15 (15q26.3) and consists of 34 exons spanning a region of over 150 kb. The coding region of the *Lrrk1* mRNA encodes a protein of 2 015 amino acids with an estimated molecular weight of 250 kDa.^38^ It is believed that *Lrrk1* and *Lrrk2* in vertebrates may be derived from the same ancient gene by DNA duplication.^38^ Though LRRK1 and LRRK2 share similar structures, including the presence of LRR, and a ROC–COR domain, a serine/threonine kinase, and WD40 repeats, only LRRK1 has ankyrin-like repeats in its N terminus.^39,40^ *Lrrk1*was first identified as a mammalian growth regulatory factor in U2OS osteosarcoma cells, and the overexpression of *Lrrk1* in human HEK293 cells was found to induce cell proliferation.^41^ However, loss of *Lrrk1* in mice caused severe osteopetrosis.^3^ *Lrrk1*^−/−^ mice were born alive, with the expected Mendelian frequency at 2 weeks of age. The body length of the *Lrrk1* KO mice was slightly shorter compared to the wild-type (WT) control littermates at 4 weeks of age. Targeted disruption of *Lrrk1* resulted in the highest observed body volumetric bone mineral density (vBMD) of the 3 629 distinct gene KO lines examined by dual-energy x-ray absorptiometry (DEXA) using high-throughput screening.^3^ The vBMD of *Lrrk1* KO mice was higher than *Sost* and *c-Src* KO mice. Both *Lrrk1* KO males and females have the same elevation in total BMD as compared to WT gender-matched littermate mice, and the increase in BMD in the long bones and spines of KO mice was persistent during aging. Micro computed tomography (micro-CT) analyses revealed that the trabecular bone volume was markedly increased in 8-week old, growing *Lrrk1* KO mice, as well as, in 79-week-old aging mice due to elevated trabecular number and trabecular thickness, and reduced trabecular separation. Interestingly, long bones had a wider metaphysis, normal diaphysis, but reduced marrow cavity area. Disruption of *Lrrk1* also resulted in slightly increased cortical bone thickness in the tibia and femur shaft in young as well as aging mice due to reduced endocortical resorption as total area (diameter) was unaffected and the marrow cavity area was reduced. In contrast to markedly elevated trabecular bone volume in long bones and vertebrae, our unpublished data showed that deficiency of *Lrrk1* had only a mild effect on the skull. The calvarias from *Lrrk1* KO mice had normal total volume (TV) but 20% higher BV and 17% higher BV/TV than control mice (Figure 1). In the mandible, TV, bone volume (BV), and BV/TV in *Lrrk1* KO mice were increased 25%, 34%, and 7%, respectively. The modest skull and mandible phenotypes of *Lrrk1* KO mice compared to the long bones and the vertebra are consistent with evidence that regulation of osteoclast function is different in membranous and endochondral bone, or that the osteoclast-mediated bone remodeling is less relevant in flat bones compared to long bones or vertebrae.

Histological analyses showed that there was extensive unresorbed cartilage below the growth plate of the distal femur and proximal tibia of *Lrrk1* KO mice, and numerous tartrate-resistant acid phosphatase (TRAP)-positive osteoclasts with an increased trabecular number and trabecular bone volume. The primary spongiosa in the *Lrrk1* KO mice extended to the diaphysis and was characterized by increased mature osteoclasts, cartilage, and trabecular bone. The secondary spongiosa was very short and incomplete. The *Lrrk1* KO osteoclasts in bone contained increased amounts of pale eosinophilic cytoplasm with enlarged scattered nuclei. Histological examinations of teeth and surrounding bones at 79 weeks of age found that *Lrrk1* KO mice had normal incisors, molars, and periodontal ligaments. The turbinate bones were of normal thickness and contained prominent basophilic (reversal) lines. Consistent with the micro-CT analyses, there was modest osteosclerosis of the periodontal bone, nasal septum, and bridge of the nose. Analyses of serum chemistry values from *Lrrk1* KO and WT control littermates showed that TRAP5b, a marker of osteoclast number, was elevated both in young and older mice.

More recently, an autosomal recessive mutation of *Lrrk1* has been identified in a human patient.^42^ A partial DNA deletion in the *Lrrk1* gene caused a frame-shift mutation, resulting in the disruption of the 7th WD40 repeats, and addition of a 66-amino-acid sequence to the C terminus of the LRRK1 protein. The mutation caused a loss of LRRK1 function in osteoclasts. The clinical features of the patient were very similar to the skeletal phenotypes observed in the *Lrrk1* KO mice. The patient with a loss of function mutation had an osteosclerotic metaphyseal dysplasia, a distinctive form of osteopetrosis characterized by severe osteosclerosis confined to the metaphysis of the long and short tubular bones due to osteoclast dysfunction.^3,42^ A skeletal survey showed a normal skull; the vertebral bodies were of normal height but had mild marginal sclerosis. The ribs were slightly broad. Marginal sclerosis of the ilia and broad, and sclerotic metaphyses of the proximal femur were noted. Hand radiographs showed broad, sclerotic metaphyses of the distal radius and ulna, and sclerotic metaphyses of the metacarpals and phalanges. The skeletal phenotypes in *Lrrk1* KO mice and the clinical skeletal signs in the affected patient with an *Lrrk1* gene mutation are summarized in Table 1. The human genetic studies together with the studies in the *Lrrk1* KO mouse model strongly suggest that LRRK1 plays a critical role in regulating osteoclast function and peak bone mass.

### *Lrrk1* and *Lrrk2* expression

Drosophila and lower vertebrate organisms such as Fugu and Zebrafish have only a single *Lrrk* gene, whereas the mammalian genome contains both *Lrrk1* and *Lrrk2* genes.^40^ Biskup *et al* recently measured *Lrrk1* and *Lrrk2* mRNA in multiple organs in neonatal and adult mice, and found that expression levels of *Lrrk1* and *Lrrk2* mRNA were almost identical in the lung, heart, skeletal muscle, and lymph node.^43^ However, *Lrrk2* mRNA is more abundant in kidney and brain tissue, whereas *Lrrk1* mRNA is more abundant in the stomach, liver, small intestine, thymus, and smooth muscle. Other studies indicated that *Lrrk2* mRNA is expressed in adult rat striatum, hippocampus, cerebral cortex, sensory and sympathetic ganglia, lung, spleen, and kidney.^44^ In the developing rat striatum, *Lrrk2* transcription is first observed at postnatal day 8, followed by increased levels of expression up to 3 weeks of age. The expression level then remains constant for nearly 2 years. The time course of postnatal development of *Lrrk2* expression patterns in the striatum thus closely mirrors the postnatal development of dopamine innervation of the striatum. *Lrrk1* is also found in most tissues of the postnatal day 1 rats. It is also known to be expressed in a number of adult rat tissues including brain, adrenal gland, liver, lung, spleen, and kidney. Interestingly, although both *Lrrk1* and *Lrrk2* are expressed in the adult human cortex cerebra, hippocampus, only *Lrrk2*, but not *Lrrk1*, is expressed in the striatum.^44,45^ Strong expression of *Lrrk2* is mainly found in neurons, specifically in the dopamine receptor 1 (DRD1a) and 2 (DRD2)-positive subpopulations of the striatal medium spiny neurons.^45^ More recently, *Lrrk1* expression is found to be low in murine osteoblasts and osteocytes, but its expression is significantly increased during the late stages of osteoclast differentiation.^42^ These studies strongly suggest that the two paralogous family members may not have overlapping functions, although they have partly complementary expression patterns in the brain, as well as in certain peripheral organs including lymphatic tissues and bones. The differential expression patterns of *Lrrk1* and *Lrrk2* genes in specific cell types and developmental stages may explain in part why loss of *Lrrk1* exhibited skeletal phenotypes but not PD, and *Lrrk2* expression could not compensate for the loss of *Lrrk1* in bones.^3^

### Functional domains of Lrrk1 in osteoclasts

**Figure 2:**
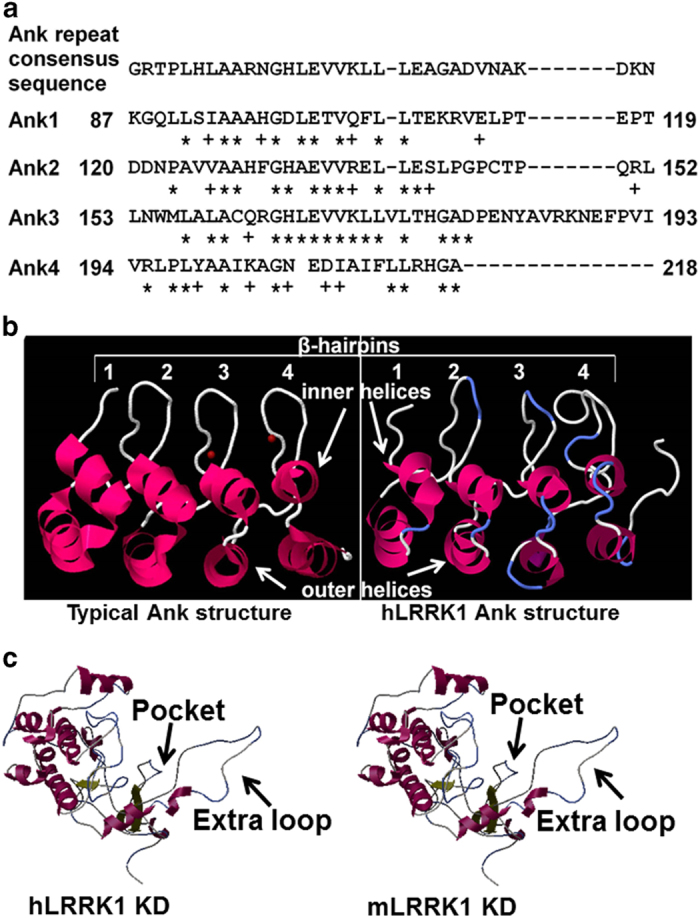
Homology models of human LRRK1 functional domains. (**a**) Sequence comparison of the human LRRK1 ankyrin (Ank) repeats with the Ank consensus sequence. (**b**) Three-dimensional (3D) structure of the ANK consensus sequence and the predicted secondary structure of human LRRK1 ANK domain. (**c**) Predicted 3D structures of the human LRRK1 and mouse LRRK1 kinase domains (KDs) with ligand-binding pockets.

The large multi-domains of LRRK1 could function as an adapter, a kinase, or a GTPase-modulating protein of focal adhesion molecules in osteoclasts. Previous studies have linked mutations in the *Lrrk2* but not the *Lrrk1* gene in humans to PD, although both *Lrrk1* and *Lrrk2* are expressed in multiple tissues including macrophage precursors.^25,40,43,46,47^ Although both LRRK1 and LRRK2 have several common functional motifs, LRRK1 but not LRRK2 contains N-terminal ANK repeats.^39,40^ Sequence analysis found that LRRK1 contains four ANK repeats that are highly identical to the ANK repeat consensus sequence. The predicted three-dimensional structure of the ANK repeat domain of human LRRK1 is similar to secondary structures of the typical ANK repeats and the Ankyrin repeat, SH3-domain, and proline-rich-region containing protein 2 (ASPP2, also known as 53BP2), which binds to p53 as a co-activator and regulates cell apoptosis.^48^ It contains functional motifs of β-hairpins, inner helices, and outer helices that can bind to important proteins involved in podosome assembly and disassembly in osteoclasts (Figures 2a and b).^48–50^ Interestingly, the integrin-linked serine/threonine kinase (ILK) also contains four ANK repeats that bind to the LIM1 domain of PINCH isoforms and ILK-associated phosphatase at the N terminus, modulating ILK protein conformation, cellular localization, F-actin remodeling, and integrin signaling.^51–53^ Inactivation of the ILK in osteoclasts in mice resulted in an increase in osteoclastogenesis both *in vitro* and *in vivo*, but ILK-deficient osteoclasts displayed a decrease in bone resorption.^53^ More recent studies involving deletion mutants of *Lrrk1* revealed that bone resorptive defects were rescued by the overexpression of LRRK1 or LRR-truncated LRRK1 in *Lrrk1*-deficient osteoclasts.^54^ However, overexpression of ANK-truncated LRRK1 failed to rescue the bone resorption function of *Lrrk1*-null osteoclasts.^54^ These data are consistent with the prediction that the ANK domain is required for LRRK1 regulation of osteoclast function. In addition, the sequences of the kinase domains in LRRK1 and LRRK2 are quite different. As an ANK repeat is one of the most common protein–protein interaction motifs, it is possible that the LRRK1 ANK repeats may mediate specific interactions of the kinase domain with its substrates such that LRRK1 has substrates that are distinct from that of LRRK2, and exercises its specific function in the bone.

Previous findings indicate that the K651A point mutation of the ROC region of LRRK1 prevents GTP binding, reduces enzymatic activity, and impairs osteoclast bone resorption.^54–56^ Recent studies also revealed that WD40 repeats are critical for LRRK1 function in human bones, as well as in mouse osteoclasts.^42,54^ Although the function of the LRRK2 WD40 domain has been well studied, little is known about the function of a putative LRRK1 WD40. It has been reported that the WD40 domain of LRRK2 can bind and sequester synaptic vesicles via interaction with vesicle-associated proteins, and a G2385R point mutation in the WD40 domain of LRRK2 correlated with a reduced binding affinity of LRRK2 to synaptic vesicles.^57^ Molecular modeling also suggests that the G2385 residue is located on the outer surface of the WD40 domain towards the C terminus, and that the substitution of the neutral and flexible glycine for a positively charged arginine at this position could interfere with inter-domain interaction, causing a 50% reduction in LRRK2 kinase activity. Consistent with this study, Jorgensen *et al* have shown that LRRK2 normally exists in a dimeric complex, and deletion of the WD40 domain prevents LRRK2 dimerization and auto-phosphorylation.^58^ A structural model of dimeric LRRK2 revealed that close contacts between the N-terminal Ankyrin and C-terminal WD40 domains, and their proximity to the kinase domain of LRRK2 regulated the kinase activity via an intramolecular mechanism.^59^ Although LRRK1 also contains a putative WD40 domain, the sequence is quite divergent from the LRRK2 WD40 domain, suggesting that the two family members may catalyze different biological substrates and interact with distinct protein partners. Recent studies from our research team have found that overexpression of WD40-truncated LRRK1 failed to rescue the bone resorption function of *Lrrk1*-deficient osteoclasts.^54^ Based on the function of the WD40 domain of LRRK2 and human genetic studies on *Lrrk1* gene mutation, it is predicted that the WD40 domain in LRRK1 is also required for LRRK1 dimerization and intermolecular or intramolecular interactions for kinase activation.^42,58^ Mutation or deletion of the WD40 domain may alter the conformation of the kinase region, leading to inactivation or disruption of protein–substrate interactions or affect subcellular localization.

### Mechanisms of Lrrk1 action in osteoclasts

The ability of the mature osteoclasts to resorb bone is largely regulated by cytoskeletal organization, and its function is dependent on actin ring or sealing zone formation. By using primary osteoclast precursors derived from WT and *Lrrk1* KO mice, recent *in vitro* osteoclast differentiation and resorptive pit formation studies revealed that *Lrrk1*-deficient monocytes can differentiate into larger, TRAP-positive multinuclear osteoclasts. Interestingly, most of the *Lrrk1*-deficient mature osteoclasts showed diffused F-actin or small actin rings in the cytoplasm, and they failed to form one large peripheral F-actin ring, and assemble sealing rings when seeded on bone slices. These flat cells remained on the bone surface, but were not associated with resorption pits, a similar phenotype also found in Wiskott–Aldrich syndrome protein (WASP)-deficient cells.^60^ Only a very small portion (<5%) of the *Lrrk1* KO osteoclasts exhibited typical but extremely large, round, and weak peripheral rings.^3^ This study indicates that dysfunction of *Lrrk1*-deficient osteoclasts contributes to the disruption of cytoskeletal rearrangement, as well as sealing ring and podosome assembly in osteoclasts.

### Potential substrates of LRRK1 kinase

Very little is known about the direct biological substrates in osteoclasts, and the mechanism underlying regulation of LRRK1 on osteoclast function. Kedashiro *et al* reported that LRRK1 regulates epidermal growth factor receptor (EGFR) trafficking by phosphorylating mouse CLIP-170 (also named as CLIP1) at threonine residue 1 384 within the waTnc motif. This promotes the association of CLIP-170 with dynein–dynactin complex formation, and the subsequent recruitment of p150^Glued^ to microtubule plus ends in HEK293 human kidney cells.^61,62^ Although changes in EGFR activation are known to influence osteoclast formation and survival,^63^ loss of *Lrrk1* in the precursors did not affect osteoclast formation and maturation. Mice with complete disruption of EGFR function exhibited a remarkable decrease in tibial trabecular bone mass with abnormalities in trabecular number and thickness due to the decreases in osteoblast number and mineralization activity, and an increase in osteoclast number.^64^ Thus, it is unlikely that an interaction of LRRK1 with EGFR will play a critical role in regulating osteoclast activity and bone mass. Though CLIP-170 is involved in cytoskeleton arrangement, male mice with disruption of CLIP-170 exhibited abnormal sperm and reduced fertility without skeletal phenotypes,^65^ whereas *Lrrk1*-deficient males showed normal fertility but severe osteopetrosis.^3^ A nonsense mutation in the human CLIP-170 gene caused the absence of CLIP-170 transcripts and protein, resulting in an autosomal recessive intellectual disability without radiographically detectable skeletal abnormalities^66^ (and personal communication). Recently, Barrera *et al* reported that LRRK1 phosphorylates DCK5RAP2 in its γ-tubulin-binding motif to promote the interaction of CDK5RAP2 with γ-tubulin. LRRK1 phosphorylation of human CDK5RAP2 at serine 140 (ggSei) is necessary for the mitotic spindle orientation.^67^ However, mice with the loss of CDK5RAP2 function exhibited small size, kyphosis, severe anemia, and neonatal death, which are not consistent with the phenotypes of *Lrrk1* KO mice that we observed.^3,68,69^ The peptide sequences of potential LRRK1 substrates from the two reports appear to lack conserved substrate motifs, and this is inconsistent with a predicted phosphor-PKCs’ substrate motif.^70^ Therefore, neither CLIP-170 nor CDK5RAP2 is likely a key biological substrate of LRRK1 in osteoclasts.

The ability of mature osteoclasts to resorb bone is largely dependent on cytoskeletal organization or sealing zone formation. The integrin αvβ3, c-Src, and Rac are key regulators of the osteoclast cytoskeleton and osteoclast activity, but do not promote osteoclastogenesis.^71–74^ Upon ligand binding, RANK and integrin αvβ3 collaborate to induce a canonical signaling pathway involving c-Src, Syk, Slp-76, Vav3, and Rac, which organize the osteoclast cytoskeleton.^75^ Deletion of any of these signaling molecules compromises the capacity of osteoclasts to remodel the cytoskeleton and resorb bone. Interestingly, the phenotypic characteristics of *Lrrk1* KO cells *in vitro*, and bone phenotypes of *Lrrk1* KO mice are very similar to *c-Src*-deficient cells or *c-Src* KO mice, but with increased severity.^76–78^ However, there is a slight difference in osteoblast mineralization and bone formation between these two gene KO strains. The mineral apposition rate and bone formation rate/bone surface were significantly reduced in Lrrk1 KO mice, although the osteoblasts appealed unimpaired because bone marrow stromal cells derived from the Lrrk1 KO mice mineralized normally *ex vivo*, and anabolic response to PTH was not altered in Lrrk1 KO mice. Mice with disruption of c-Src showed an enhanced osteoblast activity and bone formation, which also could have contributed to the increased bone mass in c-Src KO mice.^79^ Examination of the status of c-Src phosphorylation in WT and *Lrrk1* KO osteoclasts demonstrated that phosphorylation of c-Src at Tyr-527, an inactive form of c-Src, was markedly elevated in *Lrrk1*-deficient osteoclasts, whereas total c-Src protein levels were not changed between WT and KO osteoclasts. By contrast, phosphorylation of c-Src at Tyr-416, an active form of c-Src, was significantly reduced in Lrrk1 KO cells.^3^ Phosphorylation of c-Src at serine 17 has been reported to constitutively activate c-Src.^80^ However, phosphorylation of c-Src at serine 17 was not changed in *Lrrk1* KO osteoclasts. These studies suggest that c-Src might not be the direct biological substrate of LRRK1.

It is known that Csk, a nonreceptor tyrosine kinase, negatively regulates c-Src activity by phosphorylating Tyr-527 and switching the c-Src from the active open formation to an inactive closed architecture.^81,82^ There is evidence that Csk is recruited to the membrane where c-Src is in an active state through binding to Csk-binding protein.^83,84^ Mice with disruption of Csk caused embryonic lethality because of developmental arrest.^85^ However, overexpression of Csk in osteoclasts caused disorganization of the cytoskeleton, and strongly suppressed resorptive pit formation *in vitro*, whereas overexpression of kinase inactive, dominant negative Csk in osteoclasts caused increased c-Src activity, and bone-resorbing activity *in vitro* and *in vivo* assays.^86^ These observations strongly support the prediction that LRRK1 may modulate c-Src signaling pathways via interacting with Csk and modifying its function in osteoclasts.

Sun *et al*^87^ reported that a serine residue within the catalytic domain of Csk was phosphorylated and inactivated by the c-AMP-dependent protein kinase A *in vitro*. In addition, other studies also reported that protein kinase A phosphorylated Csk at serine residues and as a result inactivated Csk in the acrosome and flagellum of murine spermatozoa.^88^ Although serine 364 phosphorylation of Csk is associated with Csk auto-phosphorylation and activation in T cells,^89^ the function of serine/threonine phosphorylation in osteoclasts, and whether Csk kinase activity in *Lrrk1* KO osteoclasts is reduced remain unknown, and need to be further studied.

Although previous studies from our group have shown that LRRK1 plays a critical role in regulating osteoclast sealing zone formation, osteoclast activity, and bone resorption due to altered Tyr-527 phosphorylation of c-Src,^3^ mice with *Lrrk1* disruption exhibit a more severe osteopetrosis phenotype than *c-Src* KO mice, suggesting that LRRK1 signaling may target other signaling molecules besides the Csk/Chk/c-Src signaling pathway via post-translational modification.^3^ Several signaling pathways including integrin, nuclear factor kappa-B (NF-kB), and Src could be involved in regulating osteoclast function.^90^ Rac1/cdc42 are small guanosine triphosphatases (GTPases), which belong to the RAS superfamily of small GTP-binding proteins and are known to regulate a wide range of cellular activities, including the control of cell growth, cytoskeletal reorganization, and the activation of protein kinases. Studies have showed that double KO of *Rac1* and *Rac2* in mice caused severe metaphyseal osteopetrosis due to cytoskeleton disarrangement and osteoclast dysfunction.^73,74^ Although *Rac1/2*-deficient osteoclasts *in vitro* and *Rac1/2* KO mice exhibited bone resorption defects, the magnitude of phenotypic changes caused by lack of Rac1/2 is less severe than that of *Lrrk1*-deficient cells or *Lrrk1 KO* mice. Histomorphometric parameters of osteoblasts in Lrrk1 KO mice were similar to the adult RAC1/RAC2 KO mice. Both KO strains exhibited reduced osteoblast function and bone formation *in vivo* but the cell mineralization *in vitro* was normal, suggesting that the *in vivo* defect in osteoblast activity was not cell intrinsic. In addition, mice with deletion of *Rac1/2* in mature osteoclasts also had a normal response to PTH treatment, just as in the case of *Lrrk1* KO mice.^74,91^ Furthermore, RAC1 has been reported to interact with LRRK2 protein.^92^ These studies strongly suggest that small GTPase Rac1/Cdc42 may be direct biological substrates of LRRK1. Our recent studies have revealed that *Lrrk1* deficiency in osteoclasts resulted in reduced phosphorylation and activation of RAC1/Cdc42. *In vitro* kinase assays confirmed that LRRK1 phosphorylated RAC1-GST, and immunoprecipitation analyses found that the interaction of LRRK1 with RAC1 occurred after RANKL treatment. Overexpression of constitutively active Q61L RAC1 partially rescued the resorptive function of *Lrrk1*-deficient osteoclasts. Further studies revealed that the lack of Lrrk1 in osteoclasts led to reduced PAK1 auto-phosphorylation, catalyzed by RAC1/Cdc42 binding and activation. Interestingly, RAC1 and Cdc42 proteins bear consensus substrate motifs (RxRxxS) for PKCs and protein kinases with motifs similar to PKCs.^93^ In supporting these studies, Cdc42-deficient and Cdc42 downstream WASP KO mice also showed severe osteopetrosis phenotypes.^60,94^ Osteoclasts lacking WASP spread over a much larger surface area and are highly polykaryotic. WASP-null cells were depleted of podosomes, and failed to form actin rings at sealing zones, a phenotype similar to what *Lrrk1*-null cells exhibited.^54,60^ Based on these studies, it is likely that LRRK1 may regulate osteoclast function via modulation of phosphorylation and activation of small GTPase RAC1/Cdc42 proteins and RAC1/Cdc42 proteins may be a part of direct biological substrates of LRRK1 in osteoclasts.^54^

### Potential binding partners of LRRK1 kinase

Multiple domains of LRRK1 could also play a role as a scaffold in mediating protein–protein interactions. Recent findings have demonstrated the importance of TBC1D2-dependent Rab7 inactivation in LRRK1 regulation of autophagy formation in mouse embryo fibroblasts.^95^ Mice with disruption of *Lrrk1* were vulnerable to starvation and disrupted autolysosome formation due to a defect in lysosomal degradation during autophagy and reduced conversion of Rab7-GTP to GDP resulted from a reduction in the Rab7 GTPase-activating protein activity of TBC1D2. Indeed, Rab7, Rac1, and other Ras-like small GTPase proteins appear to act in concert to modulate ruffled border formation of bone-resorbing osteoclasts via protein–protein interactions and co-localization.^73,74,96–98^ Knockdown of Rab7 expression in cultured osteoclasts disrupted the polarization of the osteoclasts and the targeting of vesicles to the ruffled border.^96^ However, the exact role of Rab7 in the regulation of osteoclast function *in vivo* is unknown, as KO of Rab7 in mice causes embryonic lethality.^99^ Missense mutations in Rab7 in humans have been associated with Charcot–Marie–Tooth type 2B neuropathy.^100^ A pleckstrin homology domain-containing family M member 1 (Plekhm1) is believed to link to small GTPase signaling by localizing with Rab7 to late endosomal/lysosomal vesicles and functioning in vesicular transport in osteoclasts.^101^ Mice with conditional disruption of Plekhm1 in osteoclasts exhibited osteopetrosis due to abrogation of the peripheral distribution of lysosomes and bone resorption in osteoclasts.^102^ Osteoclasts derived from *Plekhm1* KO mice differentiated normally. Osteoblast number and surface were slightly decreased and the bone formation and mineral apposition rates were low in *Plekhm1*-deficient mice. However, osteoblast differentiation and bone matrix deposition *in vitro* were normal, as in the case of *Lrrk1* KO mice.^102^ Interestingly, a mutation of human Plekhm1 has been reported to cause osteopetrosis due to the defects in vesicular transport, later lysosomal, and ruffled border formation in mature osteoclasts.^101^ Full-leg radiograph from the affected patient showed cortical sclerosis of the pelvic bones and the inhomogeneous sclerosis at the metadiaphyses of the distal femora, tibiae and fibulae. These skeletal phenotypes of the Plekhm1 mutant patient seem quite different from the patient with an *Lrrk1* mutation, and *Lrrk1* KO mice that exhibited a severe osteosclerosis confined to the metaphysis of the long and short tubular bones.^3,42^ Thus, whether LRRK1 regulation of TBC1D2-dependent Rab7 inactivation and autophagy formation plays a critical role in osteoclast function will require further investigation.

More recently, it has been demonstrated that LRRK1 regulates B-cell development and activation via positively modulating CARMA1 (caspase recruitment domain, CARD, membrane-associated guanylate kinase, MAGUK, protein 1) scaffold function to activate the NF-kB cascade in B lymphocytes.^103^ B cells lacking *Lrrk1* exhibited a profound defect in proliferation and survival upon BCR stimulation, and had impaired BCR-mediated NF-kB activation and NF-kB target gene expression. The NF-kB has been shown to regulate positively osteoclast differentiation, and negatively modulate osteoblast differentiation.^104,105^ Although mice lacking NF-kB exhibited osteopetrosis due to an increase in bone resorption and bone formation *in vivo*, the phenotypic changes of osteoclasts lacking NF-kB *in vitro* were inconsistent with the osteoclast cultures derived from *Lrrk1* KO mice, ruling out involvement of NF-kB signaling in LRRK1 regulation of osteoclast function.^106^

In our previous studies, we have demonstrated that LRRK1 physically interacts with Csk in osteoclasts *in vitro*.^3^ Besides potential serine/threonine phosphorylation and inactivation of Csk, there is also a possibility that LRRK1 interacts with Csk, leading to a Csk conformation change and inactivation. In addition, interaction of LRRK1 with Csk may alter Csk membrane localization, causing reduced Csk binding to Csk-binding protein/PAG1 on the lipid rafts where c-Src is localized. Thus, we can assume that in the presence of LRRK1, Csk is on a leash, thus c-Src Y527 is not phosphorylated and the osteoclast is active. In the absence of LRRK1, active Csk phosphorylates c-Src Y527 causing c-Src inactivation and osteoclast dysfunction.

### Topologic structure of LRRK1 and LRRK2 functional domains

It has been difficult to resolve the crystal structure of LRRK2 because either full-length recombinant proteins or truncated proteins expressed in *Escherichia coli* are insoluble, unstable, or permanently bound to chaperones.^39^ Because the kinase domain is well conserved in the ROCO family of proteins across species, and ROCO4 has a sequence similarity of 47% to LRRK2, Gilsbach *et al*^107^ recently expressed the *Dictyostelium* ROCO4 kinase WT domain and corresponding PD-related mutant domains in *E. coli*, and resolved the structures of the ROCO4 kinase domain with LRRK2 inhibitor H1152. In the absence of crystal structure of the kinase domain of LRRK1 and LRRK2, the crystallography of the ROCO4 kinase domain can be used as a template for homology modeling of the LRRK2 or LRRK1 kinase domain for structure-based drug screening and structure refining.^59^ The crystal structure of the ROC domain dimer from LRRK2 has also been resolved and was used for a combination of computer-aided drug design for screening small-molecule competitors against the GTP pocket for treatment of PD.^39,108^ Little is known about the structures of LRRK1 kinase or ROC domains. Blast searches to identify suitable templates for modeling of the human LRRK1 kinase domain have led to three highly significant matches. The detected templates of transforming growth factor-beta-activated kinase 1 (TAK1), constitutive triple response 1 kinase, and mixed-lineage kinase 1 all belong to the PKC-like superfamily of serine/threonine protein kinases, and have been co-crystallized with small inhibitors.^109–111^ Although constitutive triple response 1 kinase is expressed in *Arabidopsis thaliana* and plants, its mammalian homolog MAPKKK raf plays an important role in regulating cell growth and differentiation. TAK1 and mixed-lineage kinase proteins expressed in mice and humans are also members of the MAPKKK family, and play a critical role in regulating osteoclast differentiation and bone formation.^112,113^ We have chosen TAK1 as a template to build three-dimensional structures of human LRRK1 using the ESyPred3D automated homology modeling program (Swiss Institute of Bioinformatics, Switzerland) because mice with disruption of TAK1 also show an osteopetrosis phenotype.^112,114^ Sequence analyses and homology-based protein modeling of either the human LRRK1 or mouse LRRK1 kinase domain supports that prediction that the LRRK1 kinase domain contains an extra loop in the activation site compared with the human LRRK2 kinase domain, and has a narrower active pocket for ligand binding^115^ (Figure 2c). The significant differences in the kinase domain sequence and structure of LRRK1 compared to LRRK2 strongly suggests that LRRK1 may bind to specific substrates and mediate phosphorylation of signal effectors, and that the LRRK1 kinase domain could be a drug target.

### Model for LRRK1 mechanism of action in osteoclasts

**Figure 3:**
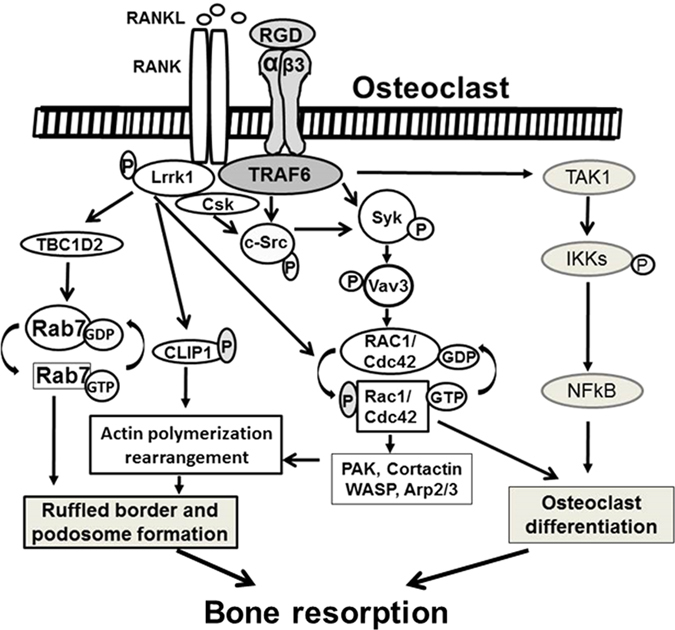
A model of the mechanism of LRRK1 action in osteoclasts. LRRK1 regulates cytoskeletal arrangement, podosome assembly, and osteoclast activity via modulating multiple signaling pathways that are triggered by various transmembrane receptors and augmenting the cellular response to a number of extracellular matrix proteins and growth factors such as integrin αvβ3, M-CSF, and RANKL.

Recent studies suggest that there is an extensive cross-talk between integrin, c-Src, and Rho signaling pathways.^90^ The osteopetrosis phenotype in *Lrrk1* KO mice are much more severe than the *c-Src* KO, *integrin beta 3* KO, or *RAC1/2* double KO mice, although these mice display partially overlapping skeletal abnormalities that are caused by defective bone remodeling and dysfunctional osteoclasts. Although integrin, Src, and Rac1 are predicted to localize in the same signaling pathway, these molecules are also regulated by multiple upstream growth factors, protein kinases, and protein phosphatases. Inactivation of only the Src/Rac1 pathway by *Lrrk1* deficiency could not explain the severe phenotypes observed in *Lrrk1* KO mice. It is, therefore, possible that LRRK1 modulates other signaling pathways besides c-Src and Rac1/Cdc42. On the basis of current publications, we propose a model of the mechanism of LRRK1 action in osteoclasts as shown in Figure 3. In this model, phosphorylation and activation of LRRK1 by membrane receptors may regulate cytoskeletal arrangement, podosome assembly, and osteoclast activity via modulating multiple signaling pathways that are also triggered by a number of extracellular matrix proteins and growth factors such as the receptor for macrophage colony-stimulating factor, EGF, tumor necrosis factors, and RANKL (receptor activator of nuclear factor kappa-B ligand). Activation of integrin αvβ3, EGF, macrophage colony-stimulating factor receptor, and RANK stimulates Syk-mediated GTP binding to Rac1/Cdc42 via the phosphorylated Vav3 in osteoclasts, activate downstream factors, and promote cytoskeletal rearrangement. Integrin αvβ3 and RANK also control small GTPase-mediated regulation of the cytoskeletal remodeling proteins WASP that is crucial for podosome formation and osteoclast polarization.^60,116^ Although LRRK1 could directly mediate phosphorylation and activation of Rac1/cdc42 and CLIP1, it could also indirectly modulate c-Src phosphorylation via inactivating Csk, and Rab7-GDP vs Rab7-GTP conversion through TBC1D2 (tubulin-specific chaperone cofactor C1 domain family member 2), stimulating ruffled border and podosome formation. Although membrane receptors can activate NF-kB signaling pathways via TAK1/IKKs, inducing osteoclast differentiation, there is no evidence that LRRK1 influences osteoclast formation through activating IKK/NF-kB signaling pathway.

### Conclusions and future directions

There is now considerable evidence to demonstrate that LRRK1 plays a key role in regulating cytoskeletal organization and osteoclast activity. Deficiency of *Lrrk1* in mice causes a much higher BMD in the long bones and vertebrae than any other gene KO mouse lines that have been tested, which makes LRRK1 an attractive alternative drug target for the treatment of osteoporosis and osteoporotic fractures.^4^ The potential substrates in osteoclasts could be Csk, CLIP1, and Rac1/Cdc42 small GTPases. However, there are a number of other issues that remain to be addressed including: (1) are there other biological substrates in osteoclasts besides Csk, CLIP1, and Rac1/Cdc42? (2) Does LRRK1 regulate functions in other cell types such as chondrocyte differentiation and hypotrophy besides osteoclast function? (3) What are the upstream activators of LRRK1 and how is LRRK1 activated? (4) How does the LRRK1 signaling pathway interact with other known signaling pathways involved in the regulation of cytoskeletal rearrangement? (5) What is the three-dimensional crystal structure of the LRRK1 kinase domain? (6) What is structural difference between the full-length WT human LRRK1 and WD40 mutant human LRRK1 and does WD40 mutation affect the kinase conformation and activation? More information about these questions will facilitate the structure-based drug design for small molecular weight inhibitors and optimize the therapeutic strategies for prevention and treatment of osteoporosis.

## Figures and Tables

**Table 1:** Comparison of phenotypes of Lrrk1 deficiency between humans and mice

Phenotypes	Deficiency of Lrrk1 in humans	Deficiency of Lrrk1 in mice
Facial features	Normal	Normal
Body length	Reduced	Reduced
Bone marrow cavities	Reduced	Reduced
BMD	Increased	Increased
Hearing problems	(−)	No data
Mental retardation	(−)	No data
Epilepsy	(−)	No data
Fracture susceptibility	(−)	No data
Hypotonia	(+)^a^	(−)
Skull bone density	Normal, measured by X-ray radiography	Mild increased, measured by micro-CT
Vertebral endplates	Sclerosis	Sclerosis
Rib costal ends	Normal	Normal
Pelvis bone	Marginal Sclerosis	Marginal Sclerosis
Long and short tubular bones	Epiphysis, metaphysis sclerosis, and under-modeling	Epiphysis, metaphysis sclerosis, and under-modeling
Cortical bone density	Normal, measured by X-ray radiography	Slightly increased, measured by micro-CT

BMD, bone mineral density; Lrrk1, leucine-rich repeat kinase 1.

a Complicated by Duchenne muscular dystrophy.
